# A Novel Test of Pure Irrelevance-Induced Blindness

**DOI:** 10.3389/fpsyg.2019.00375

**Published:** 2019-02-21

**Authors:** Christian Büsel, Thomas Ditye, Lukas Muttenthaler, Ulrich Ansorge

**Affiliations:** Faculty of Psychology, University of Vienna, Vienna, Austria

**Keywords:** attention capture, priming, load theory, irrelevance-induced blindness, continuously cumulative meta-analysis

## Abstract

Load theory claims that bottom-up attention is possible under conditions of low perceptual load but not high perceptual load. At variance with this claim, a recent one-trial study showed that under low load, with only two colors in the display – a ring and a disk –, an instruction to process only one of the two stimuli led to better memory performance for the color of the relevant than of the irrelevant stimulus. Control experiments showed that if instructed to pay attention to both objects, participants were able to memorize both colors. Thus, stimulus irrelevance diminished the likelihood of memory for a color stimulus under low perceptual-load conditions. Yet, we noted less than optimal design features in that prior study: a lack of more implicit priming measures of memory or attention and an interval between color stimulus presentation and memory test that probably exceeded 500 ms. We took care of these problems in the current one-trial study by improving the retrieval displays while leaving the encoding displays as in the original study and found that the results only partly replicated prior findings. In particular, there was no evidence of irrelevance-induced blindness under conditions in which a ring was designated as relevant, surrounding an irrelevant disk. However, a continuously cumulative meta-analysis across past and present experiments showed that our results do not refute the irrelevance-induced effects entirely. We conclude with recommendations for future tests of load theory.

## Introduction

Visual attention, the selectivity of processing seen information, is an important research area since it is involved in so many different cognitive tasks. Thus, much research on visual attention has been conducted in the past decades and important general principles of visual attention have been identified (Duncan and Humphreys, 1989; Lavie, 1995; Wolfe and Horowitz, 2004; Theeuwes, 2018). Among these general principles are the top-down control of attention that allows the observer to select the most relevant visual information (e.g., Duncan and Humphreys, 1989) and the bottom-up capture of attention that opens the gate to the processing of any salient information, be it relevant or irrelevant (Itti et al., 1998).

One theory that seeks to explain the interplay between top-down and bottom-up attention is load theory (Lavie, 1995, 2005). According to load theory, few resources are available for the bottom-up selection of just any salient visual information if the perceptual load imposed by a currently performed task is high. In other words, bottom-up attention to currently task-irrelevant but salient visual input is observed to the degree that not a high number of relevant stimuli does already deplete and block the required resources for these bottom-up processes.

At variance with this claim of load theory, Eitam et al. (2013) used a very simple, undemanding, low-load one-trial task and non-etheless observed the blocking of bottom-up selection of irrelevant information. The authors considered their findings as a challenge to load theory. In detail, participants of Eitam et al. (2013) were instructed to focus on a ring (the “outer ring” in their terminology) or, varied between participants, on a disk surrounded by the ring (the “inner ring”). The focused item was considered the relevant stimulus. Next, participants were presented with a red disk surrounded by a yellow ring. In the following, we refer to this display as the “encoding display,” as participants were later asked to retrieve colors of the stimuli that were shown in this display. However, this retrieval task was not announced to the participants in advance of the encoding display. When participants were later asked to recognize the colors of ring and disk, those participants who had focused on the ring were better at reporting the correct ring color than the disk color. The opposite pattern was observed with participants who had focused on the disk. This was a one-trial protocol, as participants would have expected the memory task in all subsequent trials and would have hence encoded both colors (i.e., attended to both stimuli) in trials following the first unannounced memory test. That such expectancies could have undermined the rationale of the protocol was confirmed in Eitam et al. (2013) control conditions in which both disk and ring were relevant by instruction and in which participants were in general able to correctly remember the colors of both items. From their results, Eitam et al. (2013) concluded that in principle resources in their one-trial task would have been sufficient to attend to two stimuli and their respective colors at the same time, but that the relevance manipulation created a bottleneck for the processing of the irrelevant stimulus. This was not predicted by load theory. Under the experimental conditions of Eitam et al. (2013), perceptual load was low and load theory would have predicted no difference between the relevant and the irrelevant stimulus in color memory accuracy.

While this might be a valid conclusion, the procedures of Eitam et al. (2013) were not maximally sensitive to reveal residual attentional effects. The current study was therefore conducted to improve the procedures of Eitam et al. (2013) and to test if their conclusions would hold if important changes to the experimental design of the study would be made. Memory retrieval accuracy was used as the dependent variable in Eitam et al. (2013) study. Although this may be necessary to some extent (but see General Discussion for alternatives), the sensitivity of the retrieval task can be improved so as to allow more evidence of bottom-up selection of the irrelevant stimulus color. Importantly, with their button press report of stimulus colors, Eitam et al. (2013) measured a relatively explicit form of memory, but no measure of implicit forms of memory or attention such as priming (cf. Tulving and Schachter, 1990; Maljkovic and Nakayama, 1994) was used by the authors. To overcome this limitation, here, we used an eye-tracker to measure where participants directed their gaze in the retrieval display. Eye movements are strongly coupled to attention (Deubel and Schneider, 1996) and the fixations could thus provide a measure of implicit priming of attention by the repetition from the encoding display of only the encoded color but not of the novel color in the retrieval display (cf. Maljkovic and Nakayama, 1994; McPeek et al., 1999; Becker, 2008). In other words, even where an explicit report of an encoded color by button press fails, a saccade to and, hence, a fixation on a repeated color in the retrieval display might be more frequent or faster than one on a novel color, thus, revealing residual implicit memory of prior attention to a previously seen (irrelevant) color.

In addition, effects of bottom-up attention may quickly vanish across time (Theeuwes et al., 2000; van Zoest et al., 2004), possibly due to active suppression of selected irrelevant information in retrospect (cf. Gaspelin et al., 2015). Thus, the interval between encoding display and retrieval task should be short, which was not necessarily the case in the original study in which written instructions for the memory task were presented in the retrieval displays. To be exact, the interstimulus interval was 500 ms but the functional interval between the encoding display and the processing of the colors in the retrieval display might have been longer than this due to the time needed to read the instructions in the retrieval display. Here, to increase the sensitivity of the method in this respect, during the retrieval display we administered auditory rather than visual memory-task instructions. This allowed us to keep the functional interval between our implicit memory measure (i.e., focusing the eyes on and looking at items in encoding and retrieval displays) at 500 ms because with auditory instructions the eyes were free to focus on the stimuli in the retrieval display already during the instructions. This compares to the unknown time it took the participants of Eitam et al. (2013) to first read the instructions in the retrieval display before they turned to the colors in the same retrieval display.

Finally, each of our participants only retrieved and reported one color per trial. This was also different from Eitam et al. (2013) who always asked for the colors of both relevant and irrelevant items (with the respective memory tasks administered in succession). We only asked for the color of one stimulus per participant to prevent even longer intervals between encoding and retrieval and to rule out any complicated carry-over effects from one report (e.g., that of a relevant ring’s color) to the other report (e.g., that of an irrelevant disk’s color).^1^ Half of our participants reported the disk and half reported the ring, and, across participants, each of the reports varied orthogonally to the shape of the relevant stimulus. Hence, one quarter of our participants had to retrieve the correct color of the relevant ring, one quarter that of the irrelevant ring, one quarter that of the relevant disk, and a final quarter that of the irrelevant disk.

Our expectations were straightforward: If Eitam et al. (2013) were right and even two simple stimuli cannot be attended to simultaneously where participants are instructed to focus on only one of the two stimuli, we should find better retrieval of the color of the relevant stimulus than of the irrelevant stimulus. In contrast, under the perspective of load theory, resources should suffice to attend to and, thus, also to select and at least prime colors of both relevant and irrelevant stimulus. Both predictions were tested with explicit (button presses) and implicit (fixations) memory measures, respectively.

## Materials and Methods

### Participants

One-hundred and eight participants [79 female, *M*_age_ = 21.52 (*SD_age_* = 2.33)] from the University of Vienna volunteered in the present study in exchange for course credits. All participants had normal or corrected-to-normal eyesight. Upon arrival, an informed consent was obtained from all the participants. Participants’ well-being was monitored throughout the experiment, and participants could abort the experiment at any point. The present study was conducted in accordance with the Declaration of Helsinki, as well as with the guidelines of the Faculty of Psychology at the University of Vienna. The Austrian Universities Act of 2002 (UG2002, Article 30 paragraph 1) states that only medical universities or studies conducting applied medical research are required to obtain an additional approval by an ethics committee. Hence, we sought no additional ethical approval.

Since the present experiment is a conceptual replication of Eitam et al. (2013), we based the power analysis for our sample size on their reported effect sizes. As Patil et al. (2016) point out, however, both original and replication studies have several sources of variation: Samples are often drawn from different participant pools and, especially in the present study, have slight methodological and analytical differences. Additionally, original studies could report an inflated effect size, which is the reason why replications can fail to observe the same effect (cf. Button et al., 2013). In light of these factors, we aimed for a power of 0.90. In their Experiment 1a, with 97 analyzed participants, Eitam et al. (2013) reported 3% erroneous recognitions in their congruent condition and 25% erroneous recognitions in their incongruent condition. In Experiment 1b, with 54 participants included in their analysis, they reported 2 and 18% erroneous recognitions in their relevant and irrelevant conditions, respectively. We weighted these proportions with respect to the number of participants in both experiments. The resulting proportion of erroneous responses was 3 and 23% for their congruent and incongruent conditions, respectively. We imputed these proportions in G^∗^Power (Faul et al., 2007) and aiming for a power of 0.90, 49 participants per condition would have been needed (98 in total). Accounting for a loss of data, we tested ten excess participants.

A new power analysis after the exclusion of data due to artifacts and/or participants’ failure to comply with the instructions in the current study was calculated. In the case of manual responses, this led to the exclusion of four participants (*N* = 104). Assuming that Eitam et al.‘s (2013) results are representative for the true population effect size, this left us with a power of 0.92 for detecting this effect in our explicit memory measure. Fifteen participants made no fixation on either stimulus in the retrieval display (implicit memory measure; *N* = 93). This loss of data left us with a power of 0.87 to detect Eitam et al.‘s (2013) effect in our implicit memory measure.

### Apparatus

Stimuli were presented on a CRT monitor, with a refresh rate of 85 Hz. Responses were given via standard QWERTZ keyboards. Stimulus presentation and response collection were managed by the Psychophysics Toolbox (Brainard, 1997; Kleiner et al., 2007) for MATLAB (MathWorks). For the recording of fixations in the retrieval displays, we used an EyeLink 1000 (SR Research), with a temporal resolution of 1,000 Hz.

### Stimuli

**FIGURE 1:**
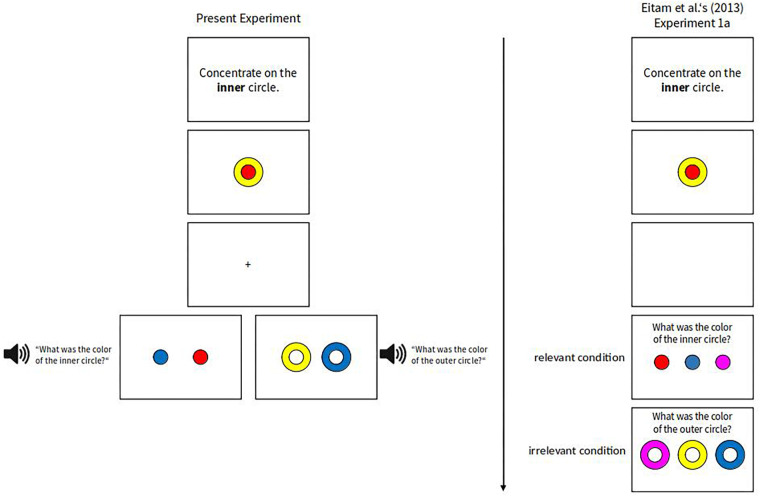
An exemplary trial from the present experiment compared to an exemplary trial from Eitam et al. (2013) Experiment 1a, with instructions to concentrate on the inner circle (i.e., the disk). The retrieval displays could either be congruent (i.e., the relevant stimulus color had to be remembered) or incongruent (i.e., the irrelevant stimulus color had to be remembered). Note that for the instructions of the participants, we used the exact same labels (i.e., *inner circle* and *outer circle*) as Eitam et al. (2013). This was done to keep instructions similar to the original. Note also that Eitam et al. (2013) asked their participants to report both relevant and irrelevant colors in successive displays, whereas in the present study we asked for only one memory report per participant, either the report of the color of the relevant item or that of the irrelevant item.

Stimuli were a disk (diameter = 1.35°) surrounded by a ring (diameter of combined disk + ring = 2.7°), with the disk being colored red (CIE L^∗^a^∗^b: 68.3/84.3/64.9) or yellow (CIE: 107.9/–15.6/90.0), and the ring in the opposite color. The two stimuli were jointly presented at screen center for 500 ms as in Eitam et al. (2013). Participants were not instructed to encode the stimuli, but as we used an unannounced memory task to test whether the two stimuli were attended to, we call this display the *encoding display*. The encoding display was followed by a retention interval showing a fixation-cross for 500 ms. This interval was the same as the official interval in Eitam et al. (2013). After the retention interval, participants were presented with a retrieval display consisting of either two rings or two disks 1.9° to the left and right from the screen center. Whether two rings or two disks were shown in the retrieval display depended on which of the stimuli from the encoding display had now to be remembered: Participants that had to remember the disk color were shown two disks and participants that had to remember the ring color were shown two rings. Together with the onset of the retrieval display, an auditory message was presented to the participants telling them to report on which side the ring (or disk) with the same color as in the preceding (i.e., encoding) display was presented. In each retrieval display, one of the stimuli had the same color as it had in the encoding display, and the other stimulus was of a novel color (i.e., blue CIE: 54.3/53.9/–103.5). Which of the two was presented on the left and which on the right was balanced across participants. The retrieval display was present until a manual response was given. An exemplary trial is shown in Figure 1.

Because stimulus relevance in the encoding display and in the retrieval display varied orthogonally, it was possible that a relevant stimulus during encoding was also relevant during retrieval – this was the *congruent condition* –, or that an irrelevant stimulus during encoding was relevant during retrieval – the *incongruent condition*. Hence, participants either had to recall the focused or the ignored stimulus color. Participants were not given a time limit for their answers in the retrieval displays. In addition to the manual responses, eye-movements were recorded.

### Procedure

A single participant was tested at a time. Upon arriving in the laboratory, participants were greeted and asked to read and sign an informed consent form. Participants were informed that the experiment also measures their eye movements, and their ocular dominance was determined. Participants were then asked to sit down at the table with the computer. The eye-tracker was positioned centrally below the monitor. After adjusting the eye-tracker, a nine-point calibration and validation was conducted. The keyboard was in front of them, on a table. Participants were shown the response keys. They were then asked to read the instructions, which were presented on the monitor. Participants were told that they could proceed with the experiment at a self-paced speed.

The instructions read – depending on the condition – “You are about to see two interlaced circles. Concentrate on the outer [or inner] circle.” Which circle should be focused upon was printed in bold and in a bigger font in order to make the relevant position more salient. After the stimuli were presented, at the time of retrieval, participants heard the recorded auditory instruction, “What color was the outer [or inner] ring? Press the left or the right key.” The auditory instructions had a duration of 4 s. The position of the correct key corresponded to the position of the stimuli in the display. The experiment was completed after participants made their responses.

### Data Pre-processing

Eye-tracking data were processed in R (version 3.4.4; R Core Team, 2018) using the package *saccades* (von der Malsburg, 2015). Data analysis was conducted with JASP (version 0.9; JASP Team, 2018) and R.

Artifacts, such as blinks, were removed from the eye-tracking data before further processing. Fixations were detected with the R package *saccades* (von der Malsburg, 2015), which uses the velocity-based saccade detection algorithm recommended by Engbert and Kliegl (2003). In this algorithm, every event between two saccades is considered a fixation. Fifteen *SD*s of the velocity distribution were chosen as the fixation detection threshold, which is also the default threshold implemented in *saccades*. Eye-tracking data were used from the same participants that were included in the analysis of the explicit memory task (i.e., four participants were excluded). Due to problems with calibration, eye-tracking data from six additional participants had to be excluded from further analyses. Hence, eye-tracking data of 98 subjects were analyzed. As the goal of this experiment was to investigate an implicit measure of item selection, we focused on fixations on the colored objects in the retrieval display (for an analysis of eye behavior in the fixation display, see Appendix). To this end, we defined regions of interest (ROIs) around the two stimuli in the retrieval display. We defined the ROIs, both for the disk and the ring condition in the retrieval display, as circles with 2.7° diameter. Only fixations starting at least 100 ms after the onset of the retrieval display were included in the analysis. Furthermore, only fixations with a duration exceeding 100 ms were analyzed.

## Results

### Explicit Memory Measure

Four participants were excluded because they did not comply with the instructions. The remaining participants’ reaction times (RTs) ranged from 0.78 to 10.79 s (and analyses of the various quartiles did not alter the results in a meaningful way). Overall accuracy of the memory report was 87.5%. Accuracies measured by percentage of correct button presses for congruent and incongruent trials were 94 and 81%, respectively. Accuracies for the different encoding and retrieval conditions are shown in Table 1.

**Table 1:** Accuracies in all possible combinations of stimulus relevance in encoding displays versus to be retrieved items.

	To be retrieved	
Relevant during encoding	Disk	Ring
Disk	100%	76.92%
Ring	84.62%	88.46%

The various combinations of stimulus relevance in the encoding versus the retrieval displays were collapsed into congruent and incongruent conditions (Table 2). A χ^2^*-*test was conducted to investigate the relationship between congruency (congruent vs. incongruent) and accuracy. The relationship between these two variables just fell short of significance, χ^2^(1) = 3.17*, p* = 0.075, continuity corrected, *Bayes Factor 10* (*BF_10_*) = 2.852. According to Kass and Raftery (1995), Bayes factors up to 3.2 are “not worth more than a bare mention” (p. 777). As can be seen from Table 1, if anything, predictions based on Eitam et al. (2013) were borne out in the disk conditions. A planned proportions test revealed that the difference between congruent and incongruent trials in the disk condition was, indeed, significant, χ^2^(1) = 6.58, *p* = 0.009. In contrast, in the ring conditions, there was no difference between memory accuracy for congruent versus incongruent conditions, χ^2^(1) = 0.27, *p* = 0.68.

**Table 2:** Participants’ performance collapsed into congruent and incongruent conditions.

	Congruency		
Correct	Congruent	Incongruent	Total
Correct	49	42	91
Incorrect	3	10	13
Total	52	52	104

### Implicit Memory Measure

Ninety-three participants fixated at least one of the two colored stimuli during its presentation in the retrieval display. As can be seen in Table 3, similar to the explicit memory performance there were quantitatively more fixations on relevant than irrelevant ROIs. This difference was—in contrast to the explicit memory performance—slightly more pronounced in the ring condition. As with participants’ accuracy of manual responses, we collapsed the various combinations of encoding versus retrieval displays into congruent and incongruent conditions (Table 4).

**Table 3:** Accuracy of first fixations on regions of interest around disk or ring in all possible combinations of relevance during encoding versus retrieval displays.

	To be retrieved	

Relevant during encoding	Disk	Ring
Disk	81%	78%
Ring	78%	90%

**Table 4:** Participants’ first fixations on a ROI collapsed into congruent and incongruent conditions.

	Congruency		
First ROI fixation	Congruent	Incongruent	Total
Correct ROI	40	36	76
Incorrect ROI	7	10	19
Total	47	46	93

**FIGURE 2:**
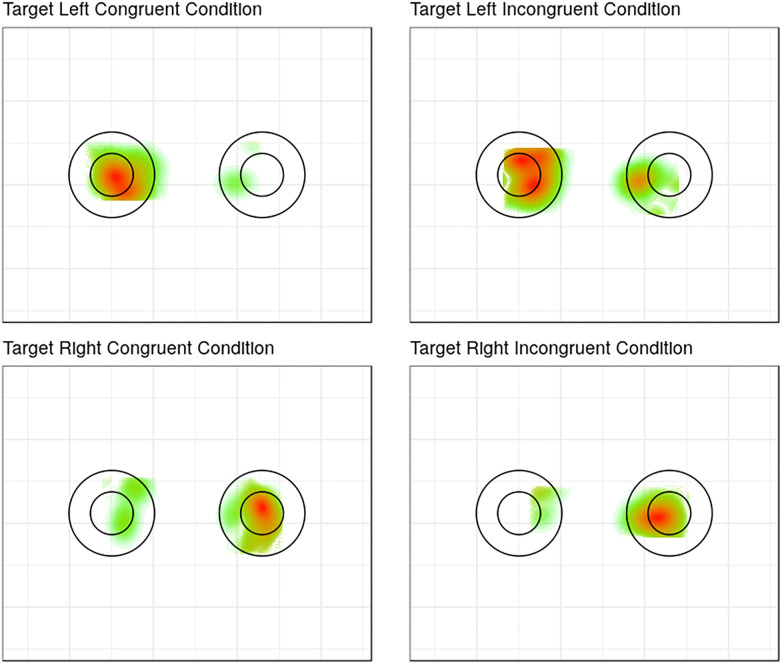
A fixation heat map indicating the density of fixations (from red = more to green = less fixations) in the respective ROIs, depending on whether a trial was congruent or not and whether the target appeared on the left or on the right. Even though both the disk and the circle are schematically shown, the ROIs were the same for the disk and the ring.

A χ^2^*-*test was conducted to investigate the relationship between congruency (congruent vs. incongruent) and accuracy of participants’ first fixation on a relevant ROI in the retrieval display (Figure 2). The relationship between these two variables was not significant, χ^2^(1) = 0.343*, p* = 0.56, continuity corrected, *BF_10_* = 0.456. This Bayes Factor suggests that the null hypothesis (i.e., there is no difference between the accuracies of first fixations in congruent and incongruent conditions) is more likely than the alternative hypothesis (the accuracy of first fixations is not equal in congruent and incongruent conditions). Planned proportions tests revealed that the difference between the proportions of correct fixations in congruent versus incongruent trials was neither significant in the disk condition, χ^2^(1) = 0.05*, p* = 0.83, nor in the ring condition, χ^2^(1) = 1.22*, p* = 0.27.

We further looked at the differences in time it took from the onset of the retrieval display and the first fixation of the correct ROI. Participants took between 177 ms and 5,128 ms to fixate the correct ROI. There was no significant difference in the latencies of the first correct fixations between congruent and incongruent trials, *t*(88) = 1.06, *p* = 0.29.

### Continuously Cumulating Meta-Analysis

It has recently been suggested that continuously cumulating meta-analyses (CCMA) provide a powerful tool to increase the precision of estimated effect sizes and counteract publication bias (Braver et al., 2014; Goh et al., 2016). CCMA essentially is an ordinary meta-analysis. Reported effects are aggregated and weighted according to their precision, which is a function of sample size (i.e., larger sample sizes yield a more precise estimate of the population effect size). CCMA refers to the idea that the already existing evidence for an effect is combined with new evidence in a continuous manner in order to increase the population effect size precision with every new publication of empirical data. Hence, CCMA provides the optimal tool to improve precision of the true size of the effect of irrelevance-induced blindness in this – or sufficiently similar – experimental protocols.

In the present experiment, we failed to conceptually replicate effects reported by Eitam et al. (2013). We chose an appropriate sample size based on the reports of Eitam et al. (2013), and even though we modified the original experimental design, we aimed at detecting the same effect as Eitam et al. (2013). Therefore, in addition to our experiment, we included both Experiments 1a and 1b from Eitam et al. (2013). (Experiment 2 of Eitam et al. (2013). was not included because it was run as a control experiment, in which participants were instructed to concentrate on both the disk and the ring. Therefore, by design, both disk and ring were task-relevant.) For the present CCMA, we collapsed data from the manual recognition responses across disk and ring conditions. We computed the logarithm of the respective studies’ odds ratios (*OR*). Our *OR*s indicate odds of a correct color indication in congruent compared to incongruent conditions. We computed the logarithm of the *OR*s because a null-effect would receive the value 0, a positive sign would be indicative of a positive influence of congruency between initial instructions and memory instruction on the memory performance, and a negative sign of a negative influence of congruency on memory measure performance.

**FIGURE 3:**
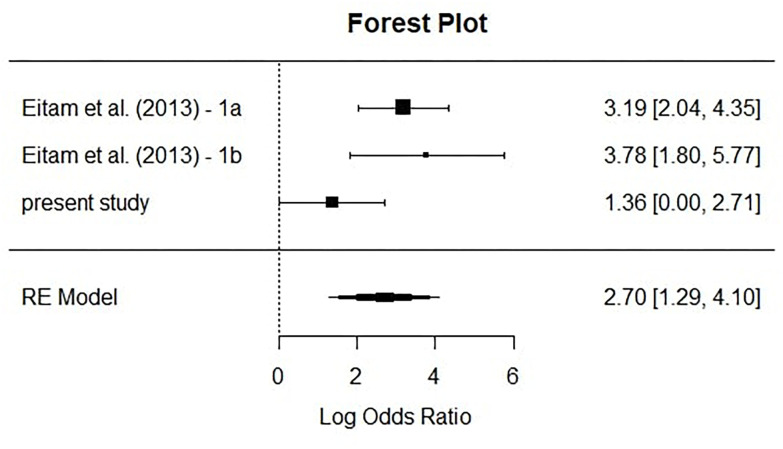
Forest plot depicting effect sizes from the single studies. The square shows the effect sizes and is larger for more precise studies (i.e., larger sample sizes) and smaller for less precise studies (i.e., smaller sample sizes). Whiskers represent the CIs of the respective studies. The diamond at the bottom depicts the accumulated and weighted effect size.

Since all the experiments included in the present CCMA (see Table 5) aimed at finding the same effect, but utilized slightly different experimental designs, we decided to compute a random-effect model. This model does not assume that the only variance between yielded effect sizes stems from sampling variance, but also from other sources such as differences in designs (cf. Borenstein et al., 2009). The results from the CCMA can be seen in Figure 3.

**Table 5:** Studies included in the present cumulative meta-analysis.

Study	*N*	*logOR*	*var(logOR)*
Eitam et al. (2013) – 1a	97	3.19	0.35
Eitam et al. (2013) – 1b	54	3.78	1.02
Present study	104	1.36	0.48

*The logarithm of the individual studies’ Odds Ratios (logOR) and their respective variances were subjected to meta-analysis.*

As can be seen in Figure 3, the accumulated and weighted effect size (the diamond) is smaller than the originally reported effect sizes of Eitam et al. (2013). However, the meta-analytical estimate is still highly significant, with *logOR* = 2.7, *p* < 0.001.

Another valuable insight is that, even though we scarcely missed to yield a significant result in manual recognition performance of the current study, this cannot be attributed to a lack of precision by our study (see CIs in Figure 3). This CCMA, however, is concerned with the *overall* influence of relevance on inattentional blindness. Yet, if anything, we were able to replicate congruency effects in the manual recognition performance for the disk instruction conditions only. This suggests that the influence of relevance on inattentional blindness is more complex than initially suspected.

## Discussion

In the present study, we aimed at conceptually replicating the findings of Eitam et al. (2013), with a more sensitive methodology of residual memory for the irrelevant colors. In the explicit measure (i.e., the participants’ manual responses), we only found significantly higher accuracies for relevant than for irrelevant objects in the disk condition but not in the ring condition. The same quantitative pattern was not significant in our implicit memory measure – the first fixations on repeated versus novel colors in the retrieval display. Hence, there was a difference for explicit, but not for implicit memory of relevant versus irrelevant objects in the disk condition. Yet, there was no difference between relevant and irrelevant objects in the ring condition, neither in the explicit nor in the implicit memory measure. Jointly, these results, thus, at best partially confirmed the conclusions of Eitam et al. (2013).

What might have created a performance difference between disk and ring conditions in the explicit memory measure? One possibility is that in the encoding displays, the disks were presented more centrally than the rings and, thus, even more in the line of gaze, as the gaze tends to center on the screen (cf. Tatler, 2007) and to center on an object (here: consisting of ring and disk) (cf. Nuthmann and Henderson, 2010). The other possibility is that relevant rings in the periphery of the compound stimulus may have entailed processing of enclosed central disks, but that processing of relevant disks did not entail processing of their surrounding rings to an equal degree. This might be due to the fact that only processing of the ring corresponded to processing of the stimulus as a whole, whereas processing of the disk might have corresponded to processing of only a part of that stimulus (cf. global precedence, e.g., Navon, 1977, plus more filtering in relevant-disk conditions). In line with this theoretical possibility, attention can be guided efficiently to part-whole color-color conjunctions but not to part-part color-color conjunctions (Wolfe et al., 1994).

Even if the current study does not provide clear-cut evidence against load theory, when integrating the present results with that of Eitam et al. (2013) in a CCMA, irrelevance-induced blindness under low-load conditions was confirmed in the explicit memory measure. In addition, data besides Eitam et al. (2013) are at variance with load theory, too. For example, during contingent-capture experiments, participants search for a predefined color target such as a red stimulus in the target display, and prior to the target a salient singleton cue can be presented (Folk et al., 1992). If this singleton cue has a color that is different from the color-homogeneous distractors in the cueing display, by virtue of its salience, it should capture attention in a bottom-up way, at least under low-load conditions. Yet, strikingly, if such a singleton cue has a color different from the searched-for target, it regularly fails to capture attention, too. For instance, during search for a red target, a salient green cue would not capture attention, as indicated by a lack of cueing effects (i.e., a lack of faster search for targets at a cued position than for targets presented away from the cue; Folk and Remington, 1998) but also by the absence of a cue-elicited N2pc – an electrophysiological marker of attention capture that can be measured in response to the cue alone (Eimer and Kiss, 2008; Ansorge et al., 2009; Eimer et al., 2009). This lack of bottom-up capture is found, although the salient irrelevant cue is presented together with only few non-singletons (all of the same color) and, thus, at a time of low perceptual load. This lack of bottom-up capture by target-dissimilar cues is at variance with load theory, too.

Now one could argue that what is true of perceptual load – that is, that it was low – is not true of cognitive load, at least in our single-trial experiment (if not in contingent-capture experiments, too). For example, at the time that the disk and ring were shown in the present study, participants might have reflected upon what exactly to do. This means that in our study, at the time of encoding, cognitive load could have been high. According to load theory, if anything, such increased cognitive load should have invited more, not less processing of the irrelevant stimuli. As Lavie (2005, p. 75) puts it: “Whereas high perceptual load can eliminate distractor processing, high load on ‘frontal’ cognitive control processes increases distractor processing.”^2^ Thus, the single-trial structure of our experiment, with its potentially increased cognitive load, might have invited more processing of the irrelevant stimuli, and this could have led both to relatively high accuracy rates and for equal accuracy rates for relevant *and* irrelevant colors. However, this would raise the question what would have prevented the same higher cognitive load and more similar performance for relevant and irrelevant colors in the original study of Eitam et al. (2013). To note, Eitam et al. (2013) participants were also not informed about the memory task and presented with a single trial only. A *post hoc* consideration of increased cognitive load would also not provide an explanation of the results of the contingent-capture experiments, with their explicit, simple and repeated task demands (i.e., search for color *x* and report criterion *y* from this position).

The upshot of these considerations is that some shortcomings entailed by procedures such as ours that do not measure attention to relevant versus irrelevant stimuli online but rather after a delay could always be subject to leveling processes, such as active inhibition following the initial capture of attention (Gaspelin et al., 2015). In fact, even features of initially attended-to stimuli can be quickly forgotten, a phenomenon labeled “attribute amnesia” (Chen and Wyble, 2015). Thus, the passing of time between encoding and retrieval display had the potential to distort the image of initial capture. Viewed from this perspective, the whole rationale of Eitam et al. (2013) as well as of the current study can be questioned.

## Conclusion

When using a, in theory, more sensitive experimental protocol, we failed to conceptually replicate the findings of Eitam et al. (2013) with an explicit memory test in their entirety. To be exact, in explicit memory, evidence for irrelevance-induced blindness under low-load conditions was found in the disk condition and missing in the ring condition. However, a joint CCMA across studies, collapsing across disk and ring conditions, confirmed irrelevance-induced blindness under low-load conditions. In addition, our implicit measure of memory was not in line with Eitam et al. (2013) findings, this time both in the disk and the ring condition. We also argue that tests of load theory should better be conducted with even more sensitive methods that measure the selection of relevant versus irrelevant colors closer to these stimuli (e.g., “online”) rather than by using memory performance as a measure of prior capture of attention alone.

## Data Availability

Datasets will be made available upon request from christian.buesel@univie.ac.at.

## Author Contributions

CB collected and analyzed the data. UA and TD designed the study. LM programmed the experiments and collected the data. UA and CB wrote the manuscript. All authors agreed with the final version of the manuscript.

## Conflict of Interest Statement

The authors declare that the research was conducted in the absence of any commercial or financial relationships that could be construed as a potential conflict of interest.

## Figures and Tables

**FIGURE 4:**
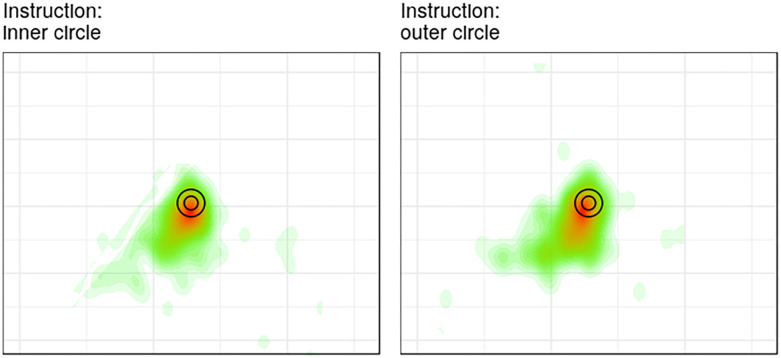
A fixation heat map showing the distribution of fixations in the encoding display (red = more to green = fewer fixations) in both the disk (left) and the ring (right) condition.

## Footnotes

1 Eitam et al. (2013) tested for an influence of the sequence of reports–that is, whether the ring (the inner circle) or whether the disk (the outer circle) had to be retrieved first–and found no significant interaction with this variable. Thus, there was seemingly nothing to worry about. However, as explained, Eitam et al. (2013) functional interval between encoding and retrieval display was relatively long in the first place, thereby, potentially masking any further delay between encoding and retrieval display. As we wanted to keep the interval between encoding and implicit measure at 500 ms with our combination of auditory instructions and eye-tracking, there was certainly no point in increasing this interval again by successive reports of both colors. In addition, Eitam et al. (2013) reported no *F* value for the non-significant interaction of reporting sequence and irrelevance-induced blindness and they did not include a power analysis demonstrating sufficient sensitivity for the interaction with their sample size. For all these reasons, we considered it safer to ask each participant for only one judgment per trial.

2 We thank the editor for this valuable idea.

## Appendix

### Dwell Times in the Encoding Display

In addition to eye-tracking data in the retrieval display, we also investigated the dwell times of fixations in the relevant regions in the encoding display. Interestingly, only 45 participants fixated either of the two colors. Dwell times at the respective regions did not vary significantly as a function of instructions (i.e., “concentrate on the inner circle” vs. “concentrate on the outer circle”). Participants looked for a similar amount of time at the ring, irrespective of whether they were instructed to concentrate on the ring or on the disk (464 ms vs. 450 ms, respectively; *t*(36.2) = 0.11, *p* = 0.92). Similarly, dwell times at the disk did not significantly differ between the two instruction conditions (497 ms for the “inner circle” instruction vs. 312 ms for the “outer circle” instruction; *t*(17.4) = 1.06, *p* = 0.30). However, that there was likely a lack of power due to only 55 participants fixating either of the colors. However, the heat map of all fixations contrasting fixations in each instruction condition also suggests that there is no significant difference in fixation distributions (here: frequencies of fixations) between the two instruction conditions (Figure 4).
