# Corrigendum: A Novel Test of Pure Irrelevance-Induced Blindness

**DOI:** 10.3389/fpsyg.2020.615123

**Published:** 2020-11-17

**Authors:** Christian Büsel, Thomas Ditye, Lukas Muttenthaler, Ulrich Ansorge

**Affiliations:** Faculty of Psychology, University of Vienna, Vienna, Austria

**Keywords:** attention capture, priming, load theory, irrelevance-induced blindness, continuously cumulative meta-analysis

In the original article, there was an error. **We stated that there was no difference in participants' correct first fixation between relevant and irrelevant rings whereas there were quantitatively more fixations on relevant than irrelevant disks. However, as can be seen in Table 3, we interchanged the ring and disk condition in the text. Similarly, in our discussion, we stated that the differences between relevant and irrelevant ring and disk conditions were identical in our explicit and implicit memory measures. However, implicit and explicit memory measures exhibit numerically opposite patterns. While the difference in the ring condition of our implicit memory measure is not statistically reliable, conjectures that are be based on the wrong rendering of the relationship between implicit and explicit memory measures might be incorrect, too**.

Corrections have been made to ***Results***, ***Implicit Memory Measure***, ***Paragraph 1***:

**Ninety-three participants fixated at least one of the two colored stimuli during its presentation in the retrieval display. As can be seen in Table 3, similar to the explicit memory performance there were quantitatively more fixations on relevant than irrelevant ROIs. This difference was—in contrast to the explicit memory performance—slightly more pronounced in the ring condition. As with participants' accuracy of manual responses, we collapsed the various combinations of encoding versus retrieval displays into congruent and incongruent conditions (Table 4)**.

The authors apologize for this error and state that this does not change the scientific conclusions of the article in any way. The original article has been updated.

